# Tracheal inflammatory response to bacterial cellulose dressing after surgical scarification in rabbits

**DOI:** 10.1016/S1808-8694(15)30597-8

**Published:** 2015-10-18

**Authors:** Angelo D’urso Panerari, Henrique Olival Costa, Flavia Coelho de Souza, Marília Castro, Leonardo da Silva, Osmar Mesquita de Sousa Neto

**Affiliations:** 1 MSc student in otorhinolaryngology at Santa Casa de São Paulo, MD, ENT, Maringá, Paraná.; 2 ENT, head and neck surgeon, PhD in otorhinolaryngology, Adjunct Professor at the Department of Otorhinolaryngology at Santa Casa de São Paulo, ENT Graduate Program Coordinator at Santa Casa de São Paulo.; 3 MSc at Escola Paulista de Medicina, Veterinarian in charge of the Instituto de Ensino e Pesquisa do Hospital Sírio Libanês.; 4 MD, Pathologist at Irmandade de Misericórdia da Santa Casa de São Paulo.; 5 PhD in otorhinolaryngology, Assistant Professor at Faculdade de Ciências Médicas da Santa Casa de São Paulo.; 6 PhD in otorhinolaryngology, Assistant Professor at Faculdade de Ciências Médicas da Santa Casa de São Paulo. Faculdade de Ciências Médicas da Santa Casa de São Paulo.

**Keywords:** cellulose, healing, rabbit, trachea

## Abstract

Exuberant scarring tissue formation is among the failure causes of tracheal stenosis surgery. Dressings that could avoid such reaction could be very helpful in these cases. Bacterial cellulose, produced by acetobacter xylinun can be useful in these cases. There are no studies in the laryngotracheal region.

**Aim:**

to assess subglottic tissue response in rabbits after scarification and placement of cellulose dressing, and comparing it to a control group.

**Study design:**

experimental.

**Materials and Methods:**

26 rabbits underwent laryngotracheal scarification, received the dressing and were compared to the control group. We established four follow up periods. Laryngotracheal specimens underwent histological exam and the results were statistically assessed.

**Results:**

the study group had statistically similar results when compared to the control group in the following parameters: vascular congestion, purulent oozing, acute inflammation, epithelial integrity, fibrous proliferation and granulous proliferation.

**Conclusion:**

we did not observe differences between the study and control groups as far as inflammation and scarring are concerned. There were no inflammatory signs associated with the use of the cellulose membrane that did no occur because of surgery.

## INTRODUCTION

Upper airway stenosis has been a troubling issue within the realm of ENT practice. The introduction of intensive care units that have allowed for improved patient survival rates has also increased the prevalence of upper way stenosis. Among the causes of laryngeal and tracheal injury are: prolonged intubation, tracheostomy, irradiation of oropharyngeal and laryngeal tumors, external trauma, idiopathic reasons. Many are the surgical procedures to treat this condition^1^, ^2^. Tracheostomies are done in 10% of the patients with brain injury and in 50-70% of the patients in a coma rated below nine in the Glasgow score. The most frequent late complication of tracheostomy is laryngeal and tracheal stenosis, a condition that affects about 15% of the patients submitted to the procedure^3^. When congenital or acquired stenosis involves the subglottic region, surgical treatment is often required due to the relevant clinical repercussions that may set in as a result of the limitation and even cessation of the patient's ability to breathe and speak.

Many are the surgical techniques adopted to repair this condition, but none has been universally effective. The procedures commonly utilized can be categorized into four groups: complete resection and termino-terminal anastomosis of the trachea, resection of the scar tissue with or without coating of the open area, incision in the stenotic site and expansion of the anterior and/or posterior wall with placement of free or pedicled grafts, and pure and simple tracheal dilation^4^, ^5^, ^6^.

All approaches have their own degrees of success and failure, but evidence points that the formation of an exuberant layer of granulation tissue on the site of treatment is one of the main reasons of failure due to restenosis^7^, ^8^, ^9^, ^10^, ^11^, ^12^.

One of the ways to prevent such tissue from growing is adopting infection preventive measures, using grafts to replace epithelium, and placing molds^2^.

Resected tissue replacement prevents the formation of an opening and the consequent formation and growth of granulation tissue, known to be a key prognostic factor in the treatment of this condition. However, surgical techniques whose aim is to cover the opening with skin or mucosal grafts end up extending surgery time and patient morbidity, as there is the additional step of having to collect the graft from another site and place it on the stenotic site 13, 14.

The possibility of using tissue that heals more naturally and is shaped in a more proper, tubular design in relation to the subglottic portion of the larynx and that does not require graft collection from another site may be quite valuable in treating this unfortunate medical condition.

Dressings for skin wounds have been considered to be, in their majority, passive devices that act as an interim protective barrier establishing a moisture-rich healing environment. A new generation of devices, designed to interact with the wound and promote new tissue formation is being developed and tested. Among such devices are the ones made from Acetobacter xylinum cellulose (Bionext®).

Bacterial cellulose is a flexible, semitransparent yellowish membrane used as a temporary substitute for skin, made up by polysaccharides synthesized by Acetobacter bacteria. It is biodegradable, non-toxic, non-pyrogenic, and sterile. This compound has been successfully used as dressing media for skin scars, burns, dermabrasion, and in skin donor sites. It has also been used as a substitute for meninges and as coating for intravascular stents to prevent circumferential stenosis in large gauge arteries.

Although there are many measures and surgical techniques to prevent exaggerated scar formation and treat stenosis, none of them has offered satisfactory results across the board. Evidence has also indicated that surgical procedures using grafts increase surgery time and patient morbidity. Therefore, an ideal dressing should prevent exaggerated scar formation, mitigate bodily response to foreign bodies, dismiss the collection of grafts from another bodily site, and not require its removal later on from the site it was implanted.

The physical and biocompatibility features offered by Bionext®, as well as ease of placement and the unlikely need to remove it afterwards may be advantageous in the cases where exaggerated scar formation has been identified.

As the use of materials with such properties has not been yet investigated in situations where exacerbated scar formation is present, and the fact that animal trials did not include its application in the trachea and subglottis, we understand it is necessary to assess tissue response to Bionext® as a temporary dressing to subglottic wounds.

This paper aims to assess subglottic tissue response in rabbits after scarification and placement of Acetobacter xylinum cellulose dressings.

## MATERIALS AND METHOD

### Rabbit selection

This study was approved by the Ethics Committee for Animal Experiments at ICAO under permit number 03/2005. The experiments were carried out at the biotherium of the Institute for Advanced ENT Sciences under the supervision of a veterinarian. All rabbits were kept in cages with free access to standardized commercial feed and water, not requiring preoperative fasting.

Twenty-six adult rabbits of both sexes were picked, with minimum weight of 1,700 grams and in good nutritional status.

The surgical procedures were performed in accordance with the regulations of the ethics committee for Experimental Surgical Techniques, federal law 6638 of May 8, 1979, and the ethical principles of experimentation postulated by the Brazilian Code for Animal Experiments (COBEA). The number of rabbits was based on the recommendations of the National Institute of Health, which deems adequate a sample of 6 to 10 animals per group when conducting preliminary surveys (http://grants.nih.gov/grants/olaw/faqs.htm).

All rabbits underwent surgery as described below.

### Surgical procedure

 

### Sample selection and size

The rabbits used in the study were adult animals of four months of age, weighing between 1,700 and 3,250 grams. In spite of the wide variation in weight, laryngeal lumen was similar in all subjects, as they were all adults.

The control group had eight rabbits, two for each follow-up stage. Sixteen subjects were enrolled in the case group, four for each follow-up stage, plus two rabbits for time zero considered as positive controls for the scarification technique.

The subjects were randomly assigned to control and case groups after undergoing scarification.

At the end of each time period the subjects were randomly picked through a draw to be assigned to the groups that would keep on being followed.

### Surgical procedure

Surgery was performed after the rabbits were anesthetized with intramuscular injections of Zoletil® (tiletamine chloridate 125/5ml and zolazepam chloridate 125/5ml), at a dose of 0.4ml/kg and Nilperidol® (fentanyl citrate + droperidol).

The animals were positioned on the surgical table in dorsal decubitus, and did not require ventilation.

Fur was performed anteriorly on the neck, extending from the jaw to the external furcula. Antisepsis was done in the anterior portion of the neck with PolvidineR. The incision was made in the mid-line from the upper border of the thyroid cartilage to approximately 0.5cm under the inferior border of the cricoid cartilage, using a 15 blade on skin and subcutaneous tissues. The incision exposed the thyroid, cricoid, and tracheal cartilages. Xylocaine at 2% associated with a vasoconstrictor was applied subcutaneously in addition to general anesthesia moments before the incisions were made.

The cricothyroid membrane was incised in its mid-line with a 15 blade, allowing the visualization of the subglottis. The opened thyroid and cricoid cartilages enabled the exposure of the laryngeal and tracheal lumen.

The subjects were kept in spontaneous ventilation through the laryngeal incision, and did not require a tracheal tube.

After the laryngeal and tracheal lumen was exposed, we used a curette to scarify the posterior subglottic mucosa, as evidenced by bleeding, extending the cuts for about 1cm in the caudal direction and 2mm to both sides laterally (Picture 1). This method was described by Branski et al. (2005) and Loewen et al. (2001) and resulted in over 50% stenosis in the rabbits operated on by the authors.

**Picture 1:**
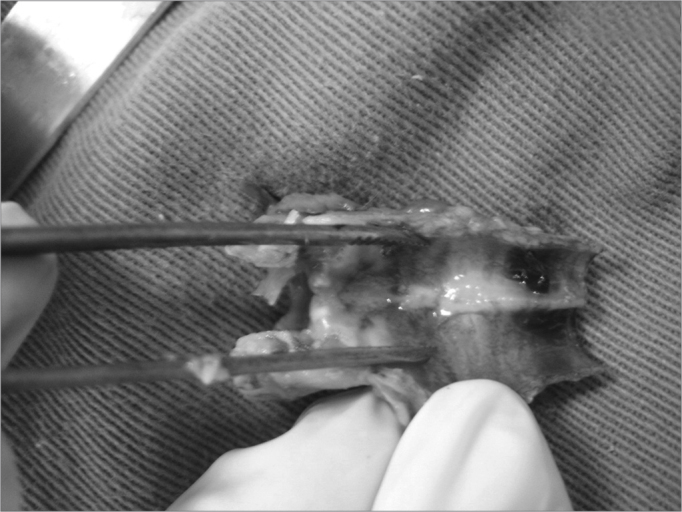
Specimen removed with tracheal mucosa resection site.

After hemostasis was achieved, a draw was made so as to assign the subjects to the following groups:

A) End procedure and assign rabbit to control group for scarification;
B) End procedure and assign rabbit to control group for healing;
C) Apply Acetobacter xylinum cellulose (Bionext®) and assign rabbit to case group for cellulose dressing.

The subjects assigned to groups A and B were then waken up from anesthesia and the procedure was called completed after the thyroid and cricoid cartilages, cricothyroid membrane, and skin were closed with Nylon 5-0 wire. Group C rabbits had a sheet of cellulose inserted and pressed against the scarified surface until it adhered to the opening in the resection plan.

After the dressing was in place, we closed the thyroid and cricoid cartilages, the cricothyroid membrane, and the skin with Nylon 5-0 wire and then woke up the subjects from anesthesia.

Clindamycin at 0.1 ml/kg was given to all rabbits when anesthesia was administered and immediately after surgery.

### Follow-up groups

The rabbits were assigned to control and case groups. Two of them were slaughtered immediately after surgery to assess the scarification produced by the adopted method before healing took place. The subjects elected for this procedure were randomly selected after scarification, so that we could go on with the procedure and place the cellulose dressing should they be assigned to the case group.

Four follow-up groups were created for the control and case groups. A draw the day before surgery was carried out to determine which subjects would be part of each of the groups.

Case group 1 had four subjects that were slaughtered one week after the completion of the procedure. Its counterpart in the control group was followed for the same time and had two rabbits.

Case group 2 had four rabbits that were slaughtered one month after the completion of the procedure. Its counterpart in the control group was followed for the same time and had two subjects.

Case group 3 had four rabbits that were slaughtered three months after the completion of the procedure. Its counterpart in the control group was followed for the same time and had two subjects.

Case group 4 had four rabbits that were slaughtered six months after the completion of the procedure. Its counterpart in the control group was followed for the same time and had two subjects. The rabbits in this group were the ones that remained from the case group.

After follow-up the subjects were pre-anesthetized and given thiopental sodium intravenously (40 mg/kg) and 2 ml of potassium chloride (KCl) at 19.1%.

After slaughtering the rabbits, we made a longitudinal incision in the neck area and removed a specimen by performing a conventional total laryngectomy combined with the removal of the upper third of the trachea. The specimen removed from such procedure was sent to the pathologist.

### Pathologist evaluation

The entire subglottic region, from the laryngeal ventricle above the free border of the vocal folds to four centimeters below it was examined (Pictures 2 and 3).

**Picture 2:**
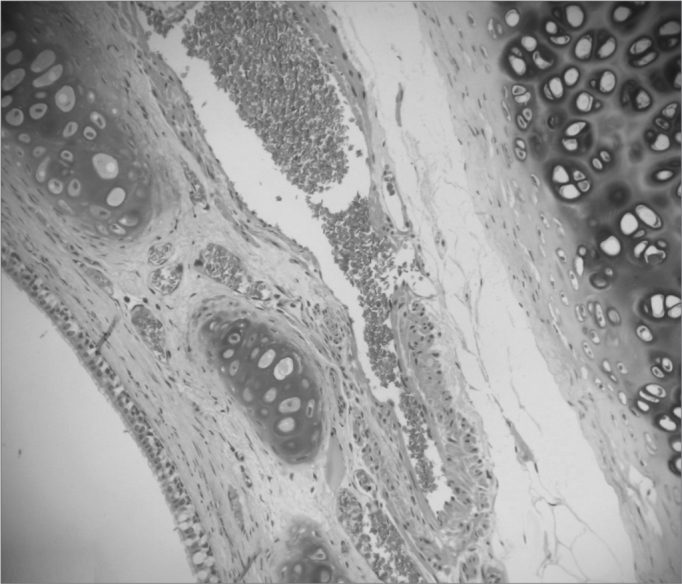
Moderate vascular congestion. HE 40X.

**Picture 3:**
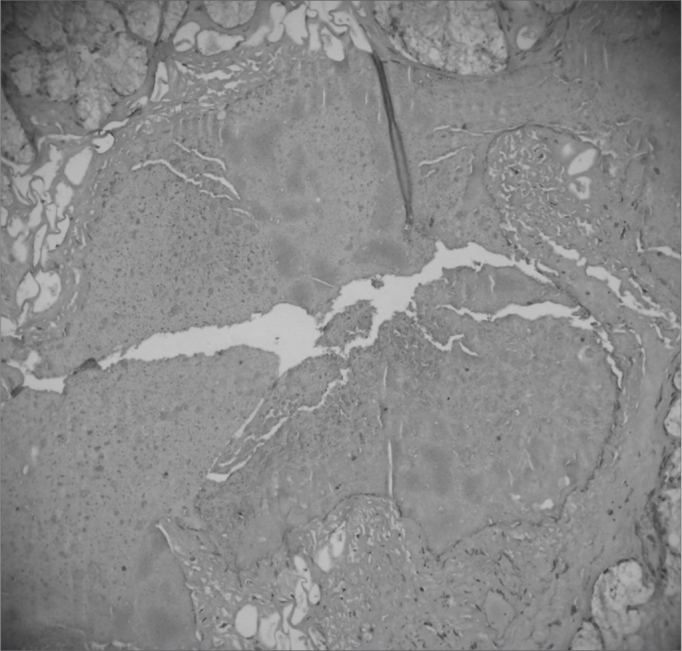
Intense vascular congestion.

The specimen removed from the subjects assigned to the positive control group for scarification was histologically assessed to verify the complete removal of the subglottic mucosa. The lesions introduced through the procedure reached the lamina propria and the perichondrium, thus evidencing the success of the method.

Degree of healing, fibrosis, and inflammation were assessed for each specimen.

The defining parameters for inflammation were the following: vascular congestion (opening of new capillaries and local venular beds, excessive dilation of existing vessels, and concentration with clustering of red blood cells inside); purulent exudates (presence of dead phagocytes or with microorganisms inside them); acute inflammation (presence of polymorphonuclears, monocytes, lymphocytes, and plasma cells, associated with overflow edema due to opening of endothelial junctions).

The defining parameters for degree of healing and fibrosis, considered healing by second intension, were the following: epithelial integrity (presence of absence of ulcerations in the coating epithelium, changes to the volume of the coating layer and/or metaplasia); fibrotic proliferation (presence of fibroblasts accumulated or permeating connective tissues); granulomatous response (accumulation of inflammatory exudate predominantly with fibroblasts and associated vascular response).

Each of the parameters was given a score as follows:

• Vascular congestion: absent (0), mild (1) and intense (2);
• Purulent exudates: absent (0), present (1).
• Acute inflammation: absent (0), present (1).
• Epithelial integrity: preserved (0), scaled (1).
• Fibrotic proliferation: absent (0), mild (1), moderate (2).
• Granulomatous response: absent (0), present (1).

Tracheal lumen was measured based on tracheal anterior-posterior and cross-sectional diameters.

All slides were prepared by the same pathologist, and all histological assessment done by one same specialist, following the standards defined by an ENT physician and a pathologist after all slides were carefully analyzed.

Both individual and average values were considered for statistical analysis.

### Statistical analysis

All results were compared between case and control groups using variance analysis and Student's T-test, for parameters with continuous values such as diameter of subglottic lumen and through Spearman's non-parametric test for non-continuous variables such as inflammation, healing, and fibrosis.

## RESULTS

After histologically analyzing the slides, the defining parameters for inflammatory condition were grouped in Chart 1, Chart 2, Chart 3, Chart 4, Chart 5, Chart 6, Chart 7, Chart 8, Chart 9, Chart 10 for control and case group rabbits, comparing the results in relation to time after surgery.

**Chart 1:**
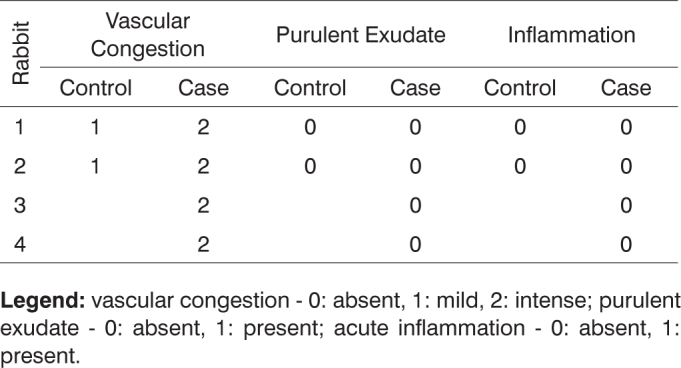
Defining parameters for inflammatory condition for case and control groups; day 7 after surgery (Group I).

**Legend:** vascular congestion - 0: absent, 1: mild, 2: intense; purulent exudate - 0: absent, 1: present; acute inflammation - 0: absent, 1: present.

**Chart 2:**
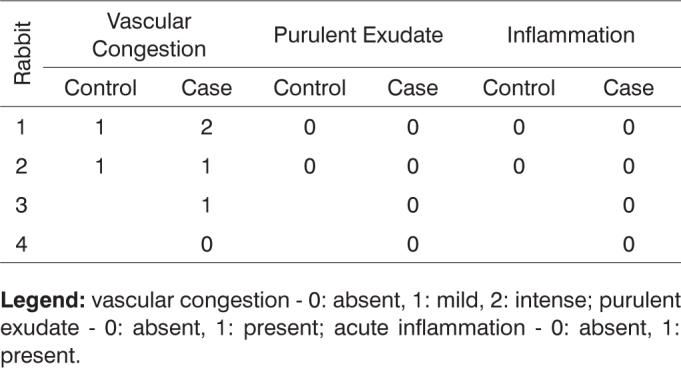
Defining parameters for inflammatory condition for case and control groups; day 30 after surgery (Group II).

**Legend:** vascular congestion - 0: absent, 1: mild, 2: intense; purulent exudate - 0: absent, 1: present; acute inflammation - 0: absent, 1: present.

**Chart 3:**
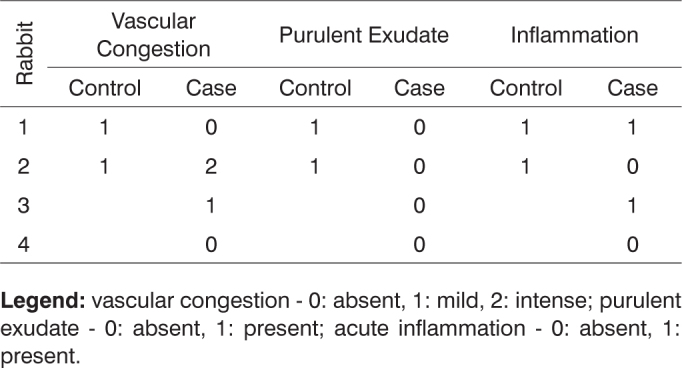
Defining parameters for inflammatory condition for case and control groups; day 90 after surgery (Group III).

**Legend:** vascular congestion - 0: absent, 1: mild, 2: intense; purulent exudate - 0: absent, 1: present; acute inflammation - 0: absent, 1: present.

**Chart 4:**
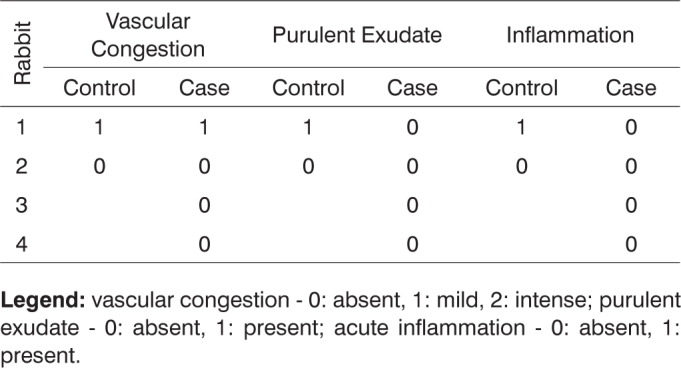
Defining parameters for inflammatory condition for case and control groups; day 180 after surgery (Group IV).

**Legend:** vascular congestion - 0: absent, 1: mild, 2: intense; purulent exudate - 0: absent, 1: present; acute inflammation - 0: absent, 1: present.

**Chart 5:**
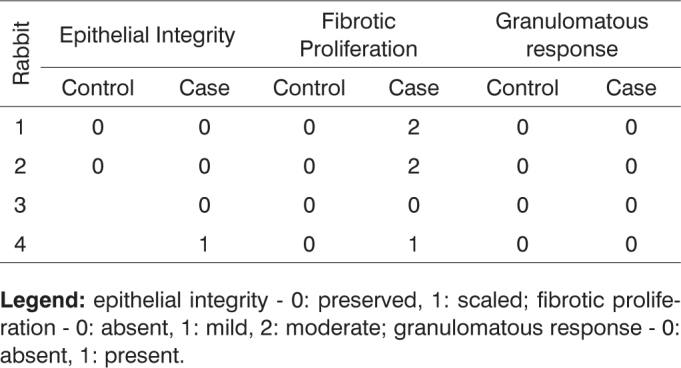
Defining parameters for degree of healing and fibrosis for control and case groups; day 7 after surgery (Group I).

**Legend:** epithelial integrity - 0: preserved, 1: scaled; fibrotic proliferation - 0: absent, 1: mild, 2: moderate; granulomatous response - 0: absent, 1: present.

**Chart 6:**
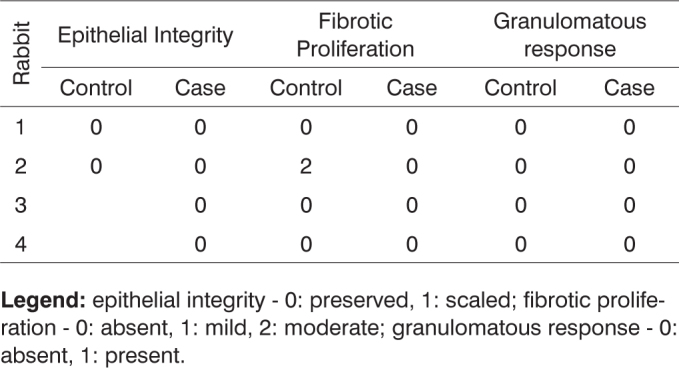
Defining parameters for degree of healing and fibrosis for control and case groups; day 30 after surgery (Group II).

**Legend:** epithelial integrity - 0: preserved, 1: scaled; fibrotic proliferation - 0: absent, 1: mild, 2: moderate; granulomatous response - 0: absent, 1: present.

**Chart 7:**
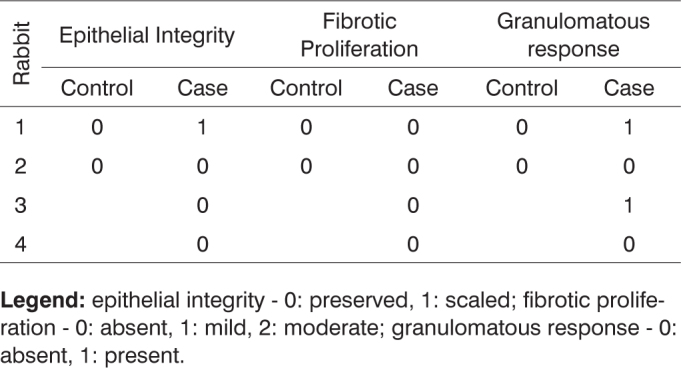
Defining parameters for degree of healing and fibrosis for control and case groups; day 90 after surgery (Group III).

**Legend:** epithelial integrity - 0: preserved, 1: scaled; fibrotic proliferation - 0: absent, 1: mild, 2: moderate; granulomatous response - 0: absent, 1: present.

**Chart 8:**
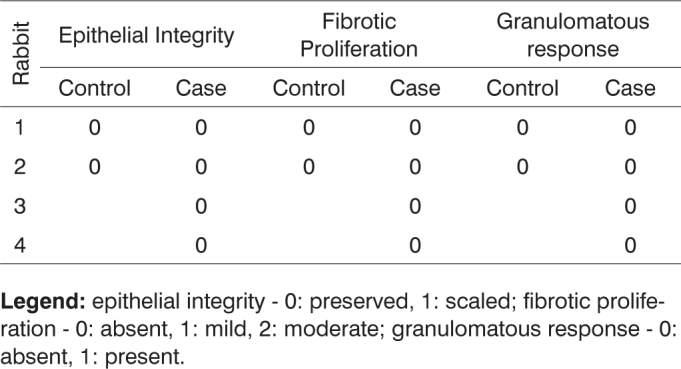
Defining parameters for degree of healing and fibrosis for control and case groups; day 180 after surgery (Group IV).

**Legend:** epithelial integrity - 0: preserved, 1: scaled; fibrotic proliferation - 0: absent, 1: mild, 2: moderate; granulomatous response - 0: absent, 1: present.

**Chart 9:**
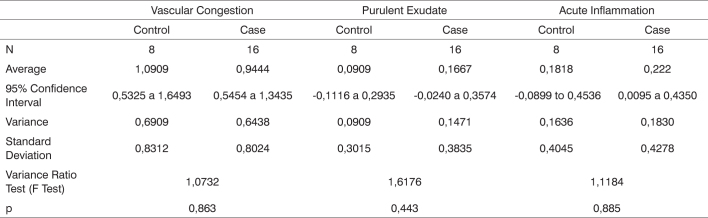
Comparison between case and control groups using Spearman's test to assess parameters vascular congestion, purulent exudate and acute inflammation.

**Chart 10:**
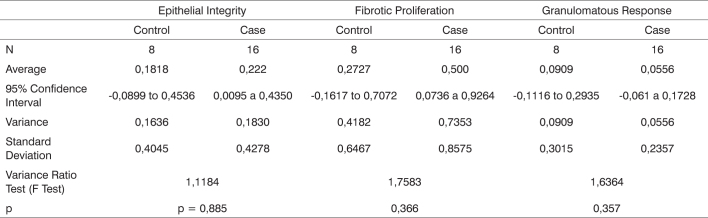
Comparison between case and control groups using Spearman's test to assess parameters epithelial integrity, fibrotic proliferation, and granulomatous response.

The results using Spearman's non-parametric test for non-continuous variables such as inflammation, healing, and fibrosis parameters are listed on Charts 6 and 7.

## DISCUSSION

The cellulose dressing has proven to be a quite effective biological dressing. Its use in the medical practice is increasing as more biologic materials are used as dressing media for injured or diseased tissues. Due to its unique structure and properties, Acetobacter xylinum-derived cellulose is a natural candidate for various medical applications where tissue restoration is the ultimate goal. This compound has been successfully used as dressing media for skin scars, burns, dermabrasion, and in skin donor sites. It has also been used as a substitute for meninges and as coating for intravascular stents to prevent circumferential stenosis in large gauge arteries^15^.

Upper airway stenosis has become an increasingly frequent problem, as technology is introduced to reduce patient death rates at the expense of increased morbidity. Many are the treatment options for laryngeal stenosis, but none have achieved universally satisfactory results. Studies have been conducted to try and identify techniques and factors that may reduce the incidence of stenosis in this area.

As cellulose dressings had been successfully used in other areas, we decided to carry out a study using rabbits to compare the healing process after subglottic scarification using Bionext® against spontaneous healing.

A scarification model was thus developed based on the methods described in the literature. Among the observed variables are:

a) Type and age of animals used
b) Subglottic scarification method
c) Number of subjects in the case and control groups

a) Type and age of animals used - adult rabbits of both sexes were used in our study, as is also the case for various others papers in the literature, as they present airways similar to those of human beings, are easy to handle, and less expensive than larger animals^16^, ^17^, ^18^, ^19^.

The subjects weighed between 1,700 and 3,250 grams. In spite of the considerable weight differences between subjects, no significant alterations were found in the subglottic region as they were adult animals, as seen in other studies in the literature^20^.

b) Subglottic scarification method - the method chosen for subglottic scarification was based in techniques described in the literature that achieved significant rates of stenosis in the subglottis and proximal trachea. The principle revolves around introducing deep enough local damage to reach the lamina propria and the perichondrium, as those are key factors in promoting intense inflammation and stenosis regardless of subject age and circumferential extension of the lesion^16^, ^11^, ^20^.

c) Number of subjects in the case and control groups - the number of subjects and their distribution within the case and control groups were based in statistical results of papers published in the literature in which subglottic scarification was performed in rabbits, consequently obtaining stenosis in 50% of the subjects on average as a result of the introduced trauma^16^, ^18^, ^19^. Given these results, we picked two subjects for the control group and four subjects for the case group, expecting that at least in one of the two rabbits there would be exacerbated scar formation, thus enabling the evaluation of the cellulose dressing in preventing exaggerated healing.

This is a pilot study. Therefore the number of subjects was reduced. We believe that in the future we may need to use a larger number of subjects to perform comparative tests between the known treatments for laryngeal and tracheal stenosis.

In spite of having used proven scarification techniques described in the literature and having introduced deep damage reaching the lamina propria and perichondrium, we did not observe healing processes accentuated enough to produce hypertrophied scars and stenosis in any of the rabbits. This fact limited the focus of the study to the analysis of scarified subglottic mucosal tissue response to Acetobacter xylinum-derived cellulose dressings.

In terms of inflammation, the following items were analyzed: vascular congestion, purulent exudate, and acute inflammation:

a) Vascular congestion (Pictures 4, 5 and 6) - in our study we found rabbits in the cellulose dressing group with intense vascular congestion within the first seven days after surgery, whereas the subjects in the control group had only mild vascular congestion. As time went by, subjects in the control group sustained mild vascular congestion in all followed groups, while the group at 180 days after surgery had one rabbit with mild vascular congestion and another rabbit without vascular congestion.

**Picture 4:**
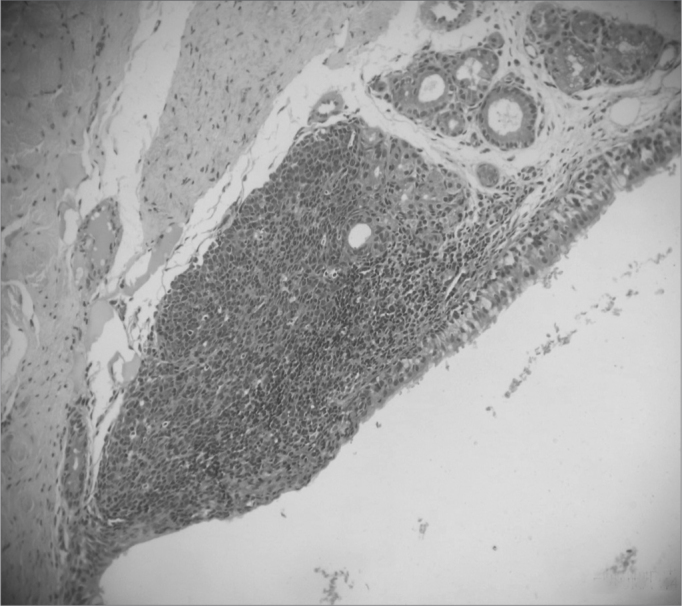
Intense acute inflammatory process. HE 40X.

**Picture 5:**
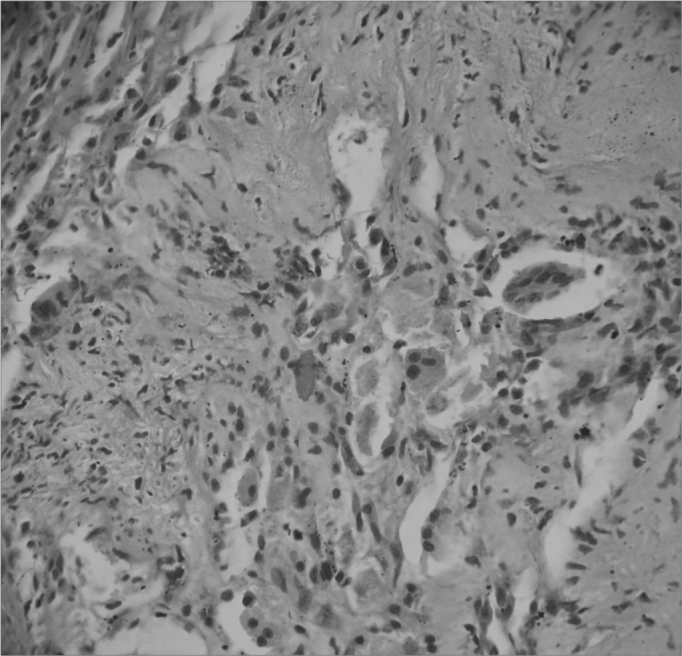
Response to foreign body in the tracheal mucosa. HE 100X.

**Picture 6:**
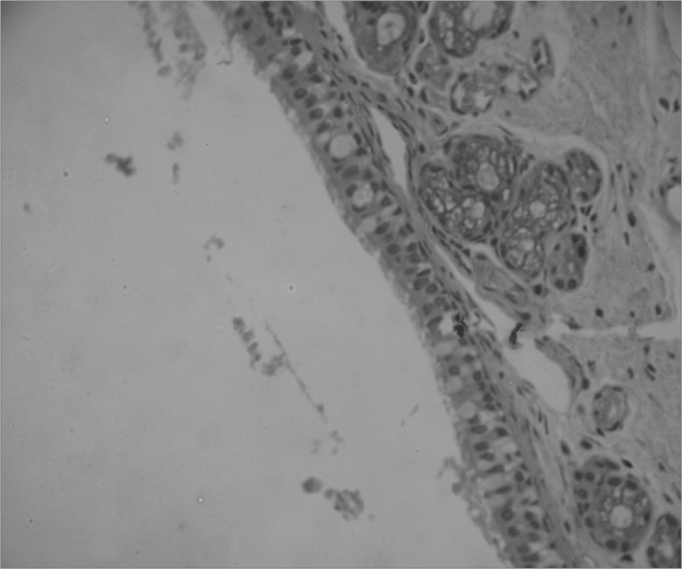
Mucosa with preserved epithelium. HE 100X.

The case group evolved to statistically similar results when compared to the control group. In the group at 180 days after surgery only one subject had mild vascular congestion, indicating that although initially the dressing may exacerbate vascular congestion and consequently inflammation, later on the process evolves to become quite similar to spontaneous healing, as observed in papers looking at graft biocompatibility in which edema and inflammation set in around the implant for the first days, only to subside later on^21^, ^22^.

This result is similar to that of other experimental studies with cellulose dressings, indicating that such a dressing does not impact healing intensity or time^23^, ^24^, ^25^, ^26^.

b) Purulent exudate - there were no statistically significant differences between the groups in time analyzed for purulent exudate when comparing control group subjects submitted only to subglottic scarification to case group subjects in whom cellulose dressings were placed, as purulent exudates did not form in any of the studied rabbits. Purulent exudate formation is a sign of exaggerated inflammatory response caused by the failure of a biomaterial in offering biocompatibility, thus leading to unsatisfactory inflammatory response, as the main aspect pertaining to biocompatibility is local tissue response^27^.

Absence of purulent exudates reveals the good response the subjects had to this dressing, avoiding exacerbated inflammation and consequent increased chance of stenosis. This may occur because of the characteristics of the cellulose produced by Acetobacter xylinum bacteria. Among them is the fact that the pores are small enough to prevent bacteria from entering the wound, but still large enough for the tissue to be properly ventilated, thus protecting it while a new mucosa is produced.

Our results are consistent with the findings or other experimental studies done on cellulose dressings in which purulent exudates were not statistically significant^23^, ^24^, ^25^, ^26^.

c) Acute inflammation (Picture 7) - our study did not reveal any statistically significant differences between inflammatory response of control group members submitted only to scarification and case group subjects submitted to scarification and cellulose dressing placement. Cellulose dressings did not exacerbate inflammation, as observed in other papers and supported by our histology findings^15^, ^26^.

Inflammatory process evolution presented no statistically significant differences in terms of intensity among the groups at 7, 30, 90, and 180 days after surgery, both for control and case groups with cellulose dressings. Only one subject in the control group at 30 days after surgery had mild inflammation.

The same was observed in the case group, with the exception of group III (90 days after surgery), as two rabbits had mild inflammation with presence of polymorphonuclears, monocytes, and lymphocytes associated to mild local edema, being such findings however not statistically significant.

Although subglottic scarification was rigorously performed as described in the literature, and the deeper layers of tissue were reached and damaged, these findings reveal that the procedure is not very effective^16^, ^17^, ^18^, ^19^, ^20^. The technique did not provide a significant inflammatory response and no exacerbation was introduced by the placement of cellulose dressings.

The cellulose dressing did not lead to exacerbated or extended inflammation due to its physical and biocompatibility characteristics, such as porosity, surface texture, consistence, and chemical properties, which altogether promote improved tissue response thus not perpetuating the inflammatory process^23^, ^24^, ^25^. One important aspect is the ability of the dressing to contain exudates, thus forming an adhesive gel that effectively encapsulates and immobilizes potentially harmful bacterial populations, which would otherwise exacerbate inflammation.

The defining parameters for degree of healing and fibrosis, considered second intension healing, are the following:

a) epithelial integrity
b) fibrotic proliferation,
c) granulomatous response.

a) Epithelial integrity - no statistically significant differences were found in the time required to detect epithelial integrity between control and case group subjects.

All rabbits in the control group had preserved epithelium at the time of the study. In the case group, one subject had scaled epithelium at seven days after surgery and another at ninety days after surgery.

Epithelial integrity is an important parameter to define scar formation and healing by second intension. Tissue regeneration in this setting is a process where intense restoration and reorganization of the various tissues involved take place, as seen in other experiments^1^, ^4^, ^28^. The absence of statistically significant differences for this parameter is relevant to stress that the cellulose dressings do not extend inflammation or promote chronic inflammatory responses, events that would lead to tissue rupture and probably extrusion f the dressing and significant epithelial alteration. This shows that cellulose dressings do not promote exacerbated tissue response, a negative impact that would possibly discourage others from using it in future studies.

b) Fibrotic proliferation: no statistically significant differences were seen when comparing presence of fibrosis in case group subjects with cellulose dressings and control group rabbits. Three subjects in the cellulose dressing group at seven days after surgery had fibrotic proliferation, being two moderate and one mild manifestation. Control group rabbits at seven days after surgery had no fibrotic proliferation. In the longer term the case group had no other case of fibrotic proliferation.

Only one subject in the control group had fibrotic proliferation at 90 days after surgery.

The presence of fibrotic proliferation in only one of the case group I rabbits and the fact that it did not evolve to inflammation leads us to believe that the dressing may initially promote fibrotic proliferation, but as time go by the process evolves quite similarly to spontaneous healing.

These findings are similar to those of other studies that used cellulose dressings in other animals and body parts that revealed the advantages such dressings offer in terms of the healing process, promoting improved healing in wounds with altered scar development^23^.

Other studies showed that Bionext® does not change the velocity or strength of the healing process, thus concluding that the dressing acts quite positively upon the healing process^20^, ^21^, ^22^, ^23^, ^25^, ^27^.

c) Granulomatous response: no statistically significant differences were verified between control and case group members with cellulose dressing through time, whether it was 7, 30, 90, or 180 days after surgery.

None of the rabbits in the control group had granulomatous response. Only two rabbits in the case group at 90 days after surgery had granulomatous response, while the rest of the subjects evolved free from such event.

Absence of granulomatous response indicates favorable response from the host tissue to the presence of cellulose dressings. According to the literature, biocompatibility responses are the reaction of the body against the presence of a foreign entity. Such reaction may progress favorably towards the formation of a thin capsule wrapping the implant and/or tissue growth inside the biomaterial, or negatively in the form of a sustained chronic inflammatory response leading to the formation of a thick fibrotic capsule, granulation, and tissue rupture with subsequent formation of an abscess or fistula, culminating with the extrusion of the dressing and neoplastic alterations.

This finding is compatible with other experimental studies conducted on the cellulose dressing. Cohn (2004) compared cellulose hydrofiber and saline drenched gauze dressings and could not find extended inflammation and consequent delayed healing.

Similar results were published by Costa (2005) when looking at skin healing in Large White pigs and comparing Bionext® to local daily dressing not observing the differences in inflammation and healing between the wounds.

Cellulose dressings did not promote granulomatous response; Bionext® is thus an inert dressing and its presence does not worsen or extend the process, as similarly found in other studies performed with this dressing^20^, ^21^, ^22^, ^23^, ^27^.

One of the main concepts in treating subglottic and tracheal stenosis is the need to cover open sites and producing increased tracheal lumen. Therefore further studies are required to better analyze the use of a biologically inert membrane that does not promote inflammation or infection, as the findings in this paper are similar to those of other studies done on cellulose dressings used in animal models.

A few advantages and possibilities may thus be listed for cellulose dressings, as follows:

• It is a viable substitute for laryngological applications;
• It implies less time and reduced surgical morbidity, as it is easy to handle and grafts do not have to be collected from other parts of the patient's body;
• Lower cost.

## CONCLUSIONS

The results produced in this study allowed the following conclusions:

• No differences were observed between case and control groups for inflammation and healing parameters.
• No signs of inflammation - other than those caused by surgical trauma - were observed in relation to the use of the cellulose membrane.
